# Integrated analysis of gene expression and metabolic fluxes in PHA-producing *Pseudomonas putida* grown on glycerol

**DOI:** 10.1186/s12934-016-0470-2

**Published:** 2016-05-03

**Authors:** Veronique Beckers, Ignacio Poblete-Castro, Jürgen Tomasch, Christoph Wittmann

**Affiliations:** Institute of Systems Biotechnology, Saarland University, Campus A1.5, 66123 Saarbrücken, Germany; Center for Bioinformatics and Integrative Biology, Biosystems Engineering Laboratory, Faculty of Biological Sciences, Universidad Andrés Bello, 8340176 Santiago, Chile; Research Group Microbial Communication, Helmholtz Centre for Infection Research, Brunswick, Germany

**Keywords:** *Pseudomonas putida* KT2440, Glycerol metabolism, Transcriptome, Metabolic flux analysis, Flux balance analysis, Elementary flux modes, Polyhydroxyalkanoates, Nitrogen and carbon limitation

## Abstract

**Background:**

Given its high surplus and low cost, glycerol has emerged as interesting carbon substrate for the synthesis of value-added chemicals. The soil bacterium *Pseudomonas putida* KT2440 can use glycerol to synthesize medium-chain-length poly(3-hydroxyalkanoates) (mcl-PHA), a class of biopolymers of industrial interest. Here, glycerol metabolism in *P. putida* KT2440 was studied on the level of gene expression (transcriptome) and metabolic fluxes (fluxome), using precisely adjusted chemostat cultures, growth kinetics and stoichiometry, to gain a systematic understanding of the underlying metabolic and regulatory network.

**Results:**

Glycerol-grown *P. putida* KT2440 has a maintenance energy requirement [0.039 (mmol_glycerol_ (g_CDW_ h)^−1^)] that is about sixteen times lower than that of other bacteria, such as *Escherichia coli*, which provides a great advantage to use this substrate commercially. The shift from carbon (glycerol) to nitrogen (ammonium) limitation drives the modulation of specific genes involved in glycerol metabolism, transport electron chain, sensors to assess the energy level of the cell, and PHA synthesis, as well as changes in flux distribution to increase the precursor availability for PHA synthesis (Entner–Doudoroff pathway and pyruvate metabolism) and to reduce respiration (glyoxylate shunt). Under PHA-producing conditions (N-limitation), a higher PHA yield was achieved at low dilution rate (29.7 wt% of CDW) as compared to a high rate (12.8 wt% of CDW). By-product formation (succinate, malate) was specifically modulated under these regimes. On top of experimental data, elementary flux mode analysis revealed the metabolic potential of *P. putida* KT2440 to synthesize PHA and identified metabolic engineering targets towards improved production performance on glycerol.

**Conclusion:**

This study revealed the complex interplay of gene expression levels and metabolic fluxes under PHA- and non-PHA producing conditions using the attractive raw material glycerol as carbon substrate. This knowledge will form the basis for the development of future metabolically engineered hyper-PHA-producing strains derived from the versatile bacterium *P. putida* KT2440.

## Background

*Pseudomonas putida* is well known for its capacity to use a wide range of carbon sources, including aromatic compounds, sugars, fatty acids and polyols [1]. The broad substrate spectrum elevates the survival rate of bacteria belonging to the genus *Pseudomonas* in comparison to other microbes, when adverse environmental conditions are present [2, 3]. In addition, *P. putida* can cope with fluctuations in nutrient availability through the accumulation of polyesters [4, 5], formed as inclusion bodies in the cytoplasm of the cell [6]. During phases of famine, degradation of these polyesters fuels the cellular demand for building blocks, redox power and energy [7]. Most *P. putida* species can synthesize a wide range of poly(3-hydroxyalkanoates) (PHAs), whereby the monomer composition of the polymer varies with carbon source and other environmental factors [8, 9]. In the past decades, PHAs have attracted considerable attention as sustainable biodegradable materials to replace oil-based polymers, especially because of their mechanical and physical properties, which are similar to conventional plastics. Meanwhile, PHAs are used at industrial scale for bags, containers, and medical devices, among others [10]. PHAs also have potential as drug carriers [11, 12]. Much effort has been poured into the development of new PHAs with tailored monomer composition [13, 14], efficient metabolically engineered strains, and fermentation processes [15, 16]. The latter aims to enhance PHA productivity and to reduce production costs, important pre-requisites for industrial production and further commercialization of PHAs. The use of industrial waste as feedstock for the synthesis of PHAs has opened a new avenue for more sustainable and cheaper microbial fermentation processes [17]. High titers of PHA have been accomplished using animal wastes [18], polyethylene terephthalate (PET) [19], and, particularly, raw glycerol from the biodiesel industry [20, 21]. Recently, we have shown that *P. putida* KT2440 is most suitable to synthesize PHA from raw glycerol among different *P. putida* strains due to reduced by-product formation under PHA-producing conditions [21]. Further studies have explored molecular details of glycerol metabolism in *P. putida* KT2440. This led to the discovery of specific regulatory genes [22, 23]. When grown in batch culture on glycerol, the gene *glpR* (PP_1074) controls utilization of the substrate. Interestingly, inactivation of this regulator leads to increased synthesis of mcl-PHA in *P. putida* KT2440 [24]. In addition, transcriptome analyses of glycerol-grown *P. putida* KT2440 indicate a mixed glycolytic and gluconeogenic pathway use, a rather complex metabolic adjustment, which strongly differs from that of cells, growing on glucose and succinate, respectively [22]. To better understand the mechanisms in *P. putida* for the production of biopolymers from glycerol, it now appears straightforward to further quantify and integrate metabolic function and regulation as well as physiological parameters in a systematic fashion. Such systems biological approaches have proven valuable to understand cellular physiology [25–27] and enable metabolic engineering approaches, with the purpose of enhancing the synthesis of target chemicals in a rational manner [28–30]. As basis for such analyses in *P. putida* KT2440, several genome-scale models have been developed from genome-annotation [31–33], unraveling the metabolic capacity of this versatile bacterium. In the present work, we explored the effect of specific growth regimes on global gene expression and carbon flux distribution of pathways of *P. putida* KT2440 in order to provide an integrated insight into its metabolic and regulatory networks during growth and PHA production on glycerol. A set of fine adjusted chemostat cultures under carbon- and nitrogen-limited conditions, and the integration of experimental and modelling data provided the basis for this systematic analysis.

## Results

### Growth physiology of *P. putida* under carbon limitation

The maintenance energy demand is of importance from both a biological and biotechnological point of view [34]. Bacterial population dynamics depend on the capacity of each member of the community to adapt to fluctuations of nutrient availability and the intrinsic metabolic energy demand for cellular functioning [35]. For biotechnological applications, microbes with low maintenance energy requirement can direct more resources for biosynthetic purposes [36], which positively impacts production performance. This, of course, also holds for *P. putida*. In this regard, the so far unknown maintenance coefficient for *P. putida* KT2440 on glycerol was quantified. For this purpose, we carefully inspected growth kinetics and stoichiometry of the bacterium, using glycerol-limited chemostat cultures at different dilution rates between 0.044 and 0.21 h^−1^. At a higher dilution rate of 0.22 h^−1^, washout of cells from the system occurred, indicating that this value approached the maximum specific growth rate of the strain on glycerol. In addition, accumulation of glycerol in the broth was observed at these elevated dilution rate. From the obtained data, specific rates for glycerol uptake (q_glycerol_) and biomass yield (Y_X/glycerol_) were calculated (Table 1).

**Table 1:** Biomass concentration, glucose uptake rate, observed yield coefficient, and carbon recovery during glycerol-limited continuous cultures of *P. putida* KT2440 for various dilution rates

D (h^−1^)	Biomass (g L^−1^)	q_glycerol_ [g (gCDW h)^−1^]	Yield_observed_ (g g^−1^)	Carbon recovery (%)
0.044	1.91	0.095	0.47	101.9
0.066	1.93	0.137	0.48	97.6
0.088	2.03	0.168	0.50	101.1
0.109	2.08	0.215	0.51	96.9
0.120	2.08	0.237	0.51	102.4
0.141	2.01	0.288	0.50	101.3
0.190	2.10	0.390	0.52	100.8
0.210	2.11	0.410	0.53	97.4

According to the Pirt model (Eq. 1), the maintenance coefficient (*m*_*glycerol*_) can be inferred from the correlation between the specific consumption rate of glycerol (*q*_*glycerol*_) and the corresponding dilution rate, i.e. the specific growth rate (*μ*) in the chemostat [37]. In addition, the relationship yields the true yield coefficient (*Y*_x/glycerol_^true^).

1 $$q_{s}^{{}} = \frac{\mu }{{Y_{x/s}^{true} }} + m_{s}$$

**Fig. 1:**
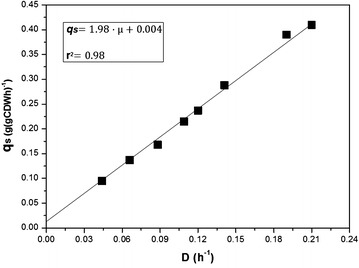
Determination of the maintenance coefficient and the true yield of glycerol-grown *P. putida* from selected chemostat experiments

As shown in Fig. 1, the specific glycerol consumption rate increased proportional with the dilution rate. Using linear regression analysis, the two coefficients were determined: *m*_*glycerol*_ = 0.039 (mmol_glycerol_) (g_CDW_)^−1^ h^−1^ and *Y*_x/glycerol_^true^ = 0.505 g_CDW_ (g_glycerol_)^−1^, respectively. Subsequently, the energy requirement for maintenance (*m*_ATP_) was derived (see Appendix) as: $m_{ATP} = \left( {0.3939 + 2.33 P/O} \right)m_{s}$. The consideration of a phosphate:oxygen (P/O) ratio of 1.75 [38], reflecting the amount of ATP formed per reduced oxygen atom during oxidative phosphorylation yielded $m_{ATP} =$ 0.175 (mmol ATP) (g_CDW_)^−1^ h^−1^, the ATP amount required for maintenance in glycerol-grown *P. putida* KT2440. Additionally, the amount of accumulated PHA and of by-products was quantified for each dilution rate. Organic acids were not detected by HPLC. The cellular PHA content was less than 3 % of the cell dry weight (CDW) at all dilution rates, which matches with natural amounts during growth on non-PHA-synthesis-related substrates [23] (Table 2). Even though there is scientific evidence that PHA can be accumulated under carbon limitation in *P. putida* strains [9, 39], this seems only the case, when fatty acids or related substrates are used as carbon substrates, where PHA precursors are inevitably generated via the β-oxidation pathway. As no by-products were detected under these carbon limiting conditions, consumed glycerol seemed to be purely metabolized into CO_2_ and biomass. As consistency check, the carbon recovery was determined from the experimental data, involving thorough balancing of carbon consumption and production, respectively. The C-mol content in the biomass was assumed to be constant, corresponding to a molecular weight of 27 g (C-mol biomass)^−1^ [40]. Within measurement accuracy, the carbon recovery was complete (96.9–102.4 %), which underlines the consistency of the data set (Table 1).

**Table 2:** Growth kinetics and stoichiometry of *P. putida* KT2440 under different growth-limiting conditions

Limitation	D (h^−1^)	Residual ammonium (mg L^−1^)	Residual glycerol (g L^−1^)	Y_X/S_ (g g^−1^)	PHA (wt%)	Y_PHA/S_ (g g^−1^)	Uptake and production rates (mmol gCDW^−1^ h^−1^)			
							Glycerol	Malate	Succinate	CO_2_
Glycerol	0.044	68.2 ± 4.8	ND < 0.1	0.47	2.5	–	1.02 ± 0.10	ND < 0.1	ND < 0.1	1.5 ± 0.1
Ammonium	0.044	ND < 0.1	19.9 ± 0.9	0.19	29.7	0.08	2.47 ± 0.15	0.04 ± 0.01	0.24 ± 0.02	3.6 ± 0.1
Glycerol	0.120	35.1 ± 2.7	ND < 0.1	0.51	2.7	–	2.60 ± 0.06	ND < 0.1	ND < 0.1	3.2 ± 0.1
Ammonium	0.120	ND < 0.1	25.7 ± 0.7	0.34	12.8	0.05	3.71 ± 0.03	0.21 ± 0.03	ND < 0.1	4.2 ± 0.1

Standard deviation (±) from at least two independent experiments

Yx/s was calculated based on the biomass, excluding PHA

*ND* not detected in the culture broth

### Growth physiology under nitrogen limitation

Next, the PHA production capacity of *P. putida* on glycerol was evaluated. In *P. putida,* PHA synthesis is driven by nitrogen limitation [9, 41]. To assess the PHA production capacity on glycerol, chemostat cultures were conducted under nitrogen limitation with glycerol as sole carbon and energy source. In order to cover different growth regimes, a low (0.044 h^−1^) and a high (0.12 h^−1^) dilution rate was adjusted, which corresponded to approximately 20 and 50 % of the maximum specific growth rate, respectively. Table 2 shows the physiological parameters, obtained under the two conditions. The biomass yield was higher at high dilution rate in comparison to the one observed at low dilution rate. The opposite result was observed for the PHA content. Here, the PHA yield reached 29.7 %wt/wt of the CDW at low dilution rate and only 12.8 %wt/wt of the CDW at high dilution rate (Table 2). The monomer composition of PHA remained rather constant (Table 3). Two organic acids, i.e. succinate and malate, were produced, when the chemostat was operated at a low dilution rate. This was not the case, when we analyzed the culture at high dilution rate, where malate was the only by-product (Table 2).

### Transcriptome analysis of *P. putida* KT2440 under carbon and nitrogen limiting conditions

To gain a more detailed insight into regulation patterns under different nutrient environments, four scenarios were selected for global gene expression analysis: N-limited growth at high (0.12 h^−1^) and low (0.044 h^−1^) dilution rate, and C-limited growth at high (0.12 h^−1^) and low dilution (0.044 h^−1^) rate, respectively (Table 2). This setup was designed to allow the extraction of changes at the transcript level originating (i) from the imposed specific growth rate and/or (ii) limitation of a specific nutrient. In Table 4, differentially expressed genes (log_2_ > 1 classified as up-regulated and log_2_ < −1 classified as down-regulated, *p* value <0.05) are listed. Obviously, a larger number of genes was affected by the imposed nutrient limitation than by the growth rate (Table 4). Genes, belonging to pathways involved in energy production and conversion, carbohydrate transport, synthesis of amino acids, nitrogen scavenging, PHA synthesis, cellular processing, and transcriptional regulation were most affected by the type of limitation (N-limiting vs. C-limiting conditions) (Table 4). On the other hand, several genes encoding proteins related to energy metabolism, transporters DNA and RNA repair and synthesis and also putative functions were differentially expressed, when *P. putida* KT2440 was challenged to different specific growth rates on glycerol. Remarkably, genes encoding signal modulators were highly overexpressed, when shifting from carbon to nitrogen limitation, independently of the set growth rate. A sensor hybrid histidine kinase PAS/PAC (PP_2664) and an integral membrane sensor (PP_2671) showed the highest change in gene expression level, which was more than 30-fold. PAS domains are important elements that sense both, fluctuation of signals in the environment and the overall energy level of the cell [42]. In addition, the LuxR (PP_2672) master gene regulator displayed a high expression level, as also did genes, allocated downstream of PAS/PAC (PP_2065-2069). Up-regulation of *PAS* and *LuxR* genes is likely related to the energetic state of the cell, as N-limiting cultures promote not only accumulation of PHAs, but also interactions between PAS domains and electron transport systems [42]. High yields of PHA diminish intracellular level of ATP [5] and to increase the NADH/NAD+ ratio [15, 43]. Probably to meet the ATP requirement under PHA-producing conditions, *P. putida* overexpressed genes, encoding for proteins of the cytochrome c-type (PP_2675, PP_43233, and PP_4324). These are essential enzymes in the electron transport chain and define the final redox state of the cell.

**Table 3:** Monomer composition of medium chain length PHA produced by *P. putida* KT2440 under nitrogen limitation

Nitrogen limitation	Monomer composition (%)					
	C6	C8	C10	C12:1	C12	C14:0
High dilution rate	<0.1	17.2	74.3	3.1	5.3	<0.1
Low dilution rate	<0.1	18.1	75.5	1.8	4.5	0.8

The data were determined by GC/MS and are given as relative molar fraction (%) of C6: 3-hydroxyexanoate, C8: 3-hydroxyoctanoate, C10:3-hydroxydecanoate, C12: 3-hydroxydodecanoate, C12:1: 3-hydroxy-5-*cis*-dodecanoate, and C14: 3-hydroxytetradecanoate

**Table 4:** Genes differentially expressed under various nutrient limitations

Metabolic function	CH vs. CL*		NH vs. NL		NH vs. CH		NL vs. CL	
	Up	Down	Up	Down	Up	Down	Up	Down
Translation, ribosomal structure and biogenesis	3	1	2	0	0	1	0	3
Transcription	1	2	0	0	4	1	8	3
Replication, recombination and repair	0	0	0	0	1	0	1	0
Energy production and conversion	2	3	2	2	14	6	12	8
Amino acid transport and metabolism	12	3	7	1	17	5	7	1
Nucleotide transport and metabolism	1	0	0	0	3	3	5	0
Carbohydrate transport and metabolism	2	2	1	0	9	6	11	8
Coenzyme transport and metabolism	5	1	3	2	3	2	9	5
Lipid transport and metabolism	1	1	1	0	2	2	1	2
Inorganic ion transport and metabolism	4	3	1	2	7	1	11	5
Secondary metabolites biosynthesis, transport, and catabolism	2	4	2	1	2	0	3	9
Cellular processing and signaling	6	14	3	1	32	9	38	6
Poorly Characterized	9	13	4	4	27	9	36	20
Total	48	47	26	13	121	45	142	70

*Carbon-limitation at high (CH) and low (CL) dilution rate, and nitrogen-limitation at high (NH) and low (NL) dilution rate are compared to extract gene modulation due to limitation (NH vs. CH and NL vs. CL) and growth rate (CH vs. CL and NH vs. NL) changes

Concerning central carbon metabolism (Table 5), increased growth rate specifically caused expression changes of genes encoding for enzymes around the phosphoenolpyruvate and pyruvate node (*pgm*, *eno*, *acoA* and *pykA*) and in the pentose phosphate (PP) pathway (*gnd*, *pgl* and *rpiA*), whereas the type of nutrient limitation rather impacted the expression of genes of the Embden-Meyerhof–Parnas (EMP) pathway (*pgi*, *fda* and *gap*-*1*), the Entner–Doudoroff (ED) pathway (*eda*) and the tricarboxylic acid (TCA) cycle (*sucA*, *sucC*, *sdhA* and *mdh*), respectively.

**Table 5:** Expression profile of genes belonging to PHA biosynthesis and central metabolic pathways in *P. putida* KT2440 under different conditions

Gene name	Locus tag	Description	Log_2_ change			
			CH vs. CL	NH vs. NL	NL vs. CL	NH vs. CH
PHA synthesis						
*phaI*	PP5008	PHA granule-associated	−0.69	−*1.10*	*1.41*	*1.00*
*phaF*	PP5007	PHA granule-associated	−0.49	−0.89	*1.11*	0.72
*phaC1*	PP5003	PHA polymerase	−0.11	0	−0.25	−0.15
*phaC2*	PP5005	PHA polymerase	−0.27	−0.29	0.48	0.46
*phaZ*	PP5004	PHA depolymerase	0.30	−0.18	0.87	0.39
*phaD*	PP5006	Transcriptional regulator	0.19	−0.24	0.50	0.07
*phaG*	PP1408	Acyl-transferase	−*1.06*	−*1.51*	*2.93*	*2.48*
Glycerol metabolism						
*oprB*	PP1019	Porin B transporter	0.76	0.37	−0.39	−0.78
*glpF*	PP1076	MIP family channel protein	0.15	0.78	−*1.28*	−0.65
*glpK*	PP1075	Glycerol kinase	0.35	0.19	−0.72	−0.88
*glpR*	PP1074	Transcriptional regulator	0.81	0.40	−0.97	−*1.39*
*glpD*	PP1073	Glycerol-3-P dehydrogenase	−0.01	−0.48	0.05	−0.42
Embden–Meyerhof–Parnas pathway						
*Glk*	PP1011	Glucokinase	0.76	0.25	−0.13	−0.65
*gltR*	PP1012	Transcriptional regulator	0.78	0.43	−0.7	−*1.05*
	PP1013	Integral membrane sensor	0.7	0.4	−*1.00*	−*1.3*
*Pgi*	PP1808	Glucose-6-phosphate isomerase	0.13	0.11	−0.10	0.13
*Fbp*	PP5040	Fructose-1,6-bisphosphatase	0.21	−0.12	−0.10	0.11
*Fda*	PP4960	Fructose-1,6-bisphosphate aldolase	−0.13	0.24	−0.10	0.5
*tpiA*	PP4715	Triosephosphate isomerase	0.82	0.47	−0.10	−0.93
*gap1*	PP1009	GAP dehydrogenase, type I	0.30	0.69	−*1.32*	−0.92
*gap2*	PP2149	GAP dehydrogenase, type II	0.14	−0.06	−0.10	0.42
*pgk*	PP4963	Phosphoglycerate kinase	0.08	0.37	−0.10	0.46
*pgm*	PP5056	Phosphoglyceromutase	0.25	0.24	−0.1	0.04
*eno*	PP1612	Phosphopyruvate hydratase	0.18	0.46	−0.1	0.07
*pyk*	PP1362	Pyruvate kinase	0.11	0.30	−0.1	0.85
Pentose phosphate pathways						
*zwf1*	PP1022	G6P dehydrogenase	*1.33*	0.39	0.85	−0.09
*zwf2*	PP4042		0.48	0.14	−0.10	−0.48
*zwf3*	PP5351		−0.23	−0.05	−0.10	0.21
*pgl*	PP1023	6-P-gluconate dehydrogenase	0.65	0.42	*1.15*	0.77
*gnd*	PP4043	6-P-gluconate dehydrogenase	0.08	−0.15	−0.1	−0.23
*gnuK*	PP3416	Carbohydrate kinase	−0.06	−0.52	−0.10	−0.7
*kguK*	PP3378	Dehydroglucokinase	−0.02	−0.63	−0.10	−0.21
*kguD*	PP3376	2-Ketogluconate 6-phosphate reductase	−0.15	−0.17	−0.10	−0.2
*rpiA*	PP5150	Ribose-5-phosphate isomerase A	−0.09	0.06	−0.10	0.06
*rpe*	PP0415	Ribulose-phosphate 3-epimerase	−0.04	0.20	−0.74	−0.5
*tktA*	PP4965	Transketolase	0.28	0.29	−0.10	0.46
*tal*	PP2168	Transaldolase B	−0.6	−0.07	−0.10	0.70
Entner-Doudoroff pathway						
*edd*	PP1010	6-Phosphogluconate dehydratase	0.9	0.22	−0.03	−0.71
*eda*	PP1024	KDPG aldolase	*1.24*	0.29	*1.65*	0.98
Pyruvate metabolism						
*acoA*	PP0555	Pyruvate dehydrogenase	*1.16*	*1.86*	−*1.03*	−0.33
*acoB*	PP0554	Pyruvate dehydrogenase	*1.5*	*1.88*	−*1.04*	−0.66
*acoC*	PP0553	Pyruvate dehydrogenase	*1.57*	*1.81*	−0.96	−0.72
	PP0545	Aldehyde dehydrogenase	0.2	0.11	−0.91	−1.01
*acsA*	PP4487	Acetyl-CoA synthetase	−0.13	0.23	−0.15	*3.56*
*accC*-*2*	PP5347	Pyruvate carboxylase	0.22	−0.27	−0.12	0.07
*ppsA*	PP2082	Phosphoenolpyruvate synthase	0.10	0.22	−0.12	0.09
*ppc*	PP1505	Phosphoenolpyruvate carboxylase	0.09	−0.06	−0.10	0.50
	PP0154	Acetyl-CoA hydrolase	0.21	0.23	*1.42*	*1.41*
TCA cycle						
*gltA*	PP4194	Citrate synthase	0.01	0.57	−0.10	*1.12*
*acnA*	PP2112	Aconitate hydratase	−0.04	−0.22	−0.10	−0.57
*acnB*	PP2339	Aconitate hydratase	−0.33	0.2	−0.10	0.74
*icd*	PP4012	Isocitrate dehydrogenase	0.38	−0.14	−0.10	−*1.97*
*sucA*	PP4189	2-Oxoglutarate dehydrogenase	0.37	0.08	−0.10	−0.17
*sucD*	PP4185	Succinyl-CoA synthetase sub alpha	0.6	0.35	−0.10	−0.13
*sucC*	PP4186	Succinyl-CoA synthetase sub beta	0.47	0.35	−0.10	0.14
*sdhA*	PP4191	Succinate dehydrogenase	0.24	0.30	−0.10	−0.04
*fumC*	PP0944	Fumarate hydratase	−*2.46*	−0.63	−*1.92*	−0.08
*mdh*	PP0654	Malate dehydrogenase	−0.79	−0.52	−0.42	−0.15
Glyoxylate shunt						
*aceA*	PP4116	Isocitrate lyase	−0.78	0.03	*3.10*	*3.91*
*glcB*	PP0356	Malate synthase	0.19	0.32	0.69	0.82

Carbon-limitation at high (CH) and low (CL) dilution rate, nitrogen-limitation at high (NH) and low (NL) dilution rate

Boldface represents a differentiated expression pattern

*p* value <0.05

### The metabolic flux profile of glycerol-grown *P. putida* is affected by the cellular environment

**Fig. 2:**
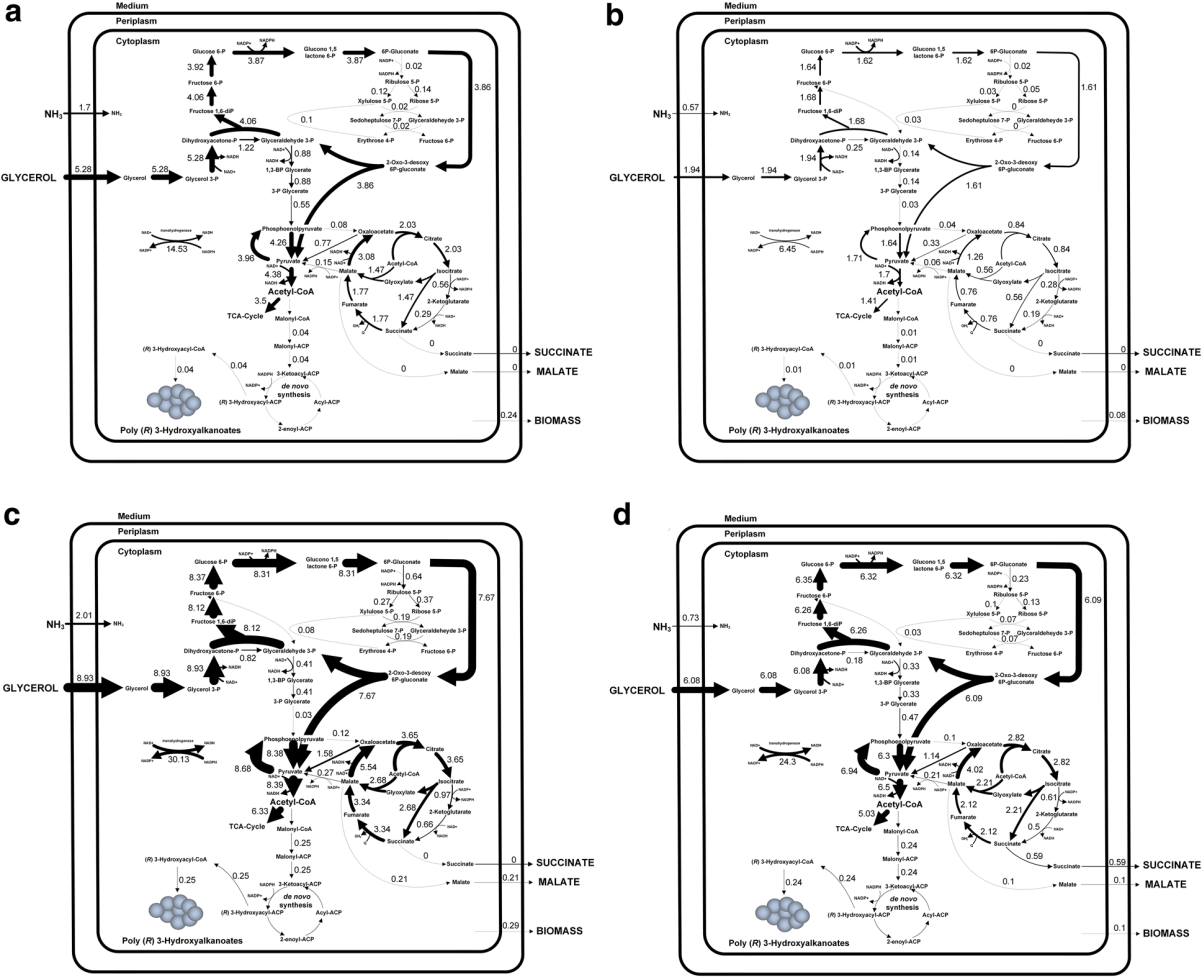
Predicted intracellular fluxes for maximized growth from flux balance analysis under nitrogen and carbon-limiting conditions, both for a high dilution rate (0.12 h^−1^) and a low dilution rate (0.044 h^−1^). Intracellular fluxes in *P. putida* under carbon limitation with a high (**a**) and a low (**b**) dilution rate and under nitrogen limitation with a high (**c**) and a low (**d**) dilution rate. All molecular fluxes are given next to the respective arrow and are given in mmol L^−1^ h^−1^, except for the biomass formation, which is given in mmol g_CDW_^−1^  h^−1^. The width of each arrow also represents the size of the corresponding flux, except for the width of the arrow belonging to the transhydrogenase reaction, which, for visualization purposes, correlates to one tenth of the corresponding flux size

Beyond the demonstrated influence of specific growth rate and nutrient limitation on the regulatory network of *P. putida* we next performed flux balance analysis to additionally evaluate the performance of the underlying metabolic network for the different scenarios. Metabolic fluxes of central carbon metabolism were inferred by flux balance analysis, based on a detailed metabolic network of *P. putida* (see Appendix), which was constrained by experimental uptake and production rates to reveal the investigated physiological states (Table 2). Hereby, the relative weight of individual pathways for biomass and PHA synthesis was deduced from the obtained fluxes. Figure 2 shows the distribution of the calculated fluxes for each condition.

As predicted by the modeling approach, *P. putida* preferentially uses the oxidative PP and the ED pathway under nitrogen limiting conditions. The corresponding flux values were 2.1- and 3.9-fold higher than that under carbon-limitation at high and low dilution rate, respectively. Due to the fact that glycerol uptake only increased by a factor of 1.7 and 3.1, respectively, not only absolutely, but also relatively more carbon was channeled through the ED pathway. Additionally, the TCA cycle was 1.8 and 3.3 times more active at high and low dilution rate, respectively, whereas the carbon partitioning at the isocitrate node, directed relatively more carbon through the glyoxylate shunt (73 and 78 % vs. 72 and 67 %, respectively). No by-product formation was detected under carbon-limitation. In contrast, a minor flux of carbon was funneled to malate and succinate synthesis from the TCA cycle under nitrogen-limiting conditions. These apparent differences in carbon partitioning were associated with a 7- and 20-fold increased PHA biosynthetic flux under nitrogen-limitation at high and low dilution rate, respectively. Concerning redox equivalents, more NADPH was needed to satisfy the increased demand under nitrogen limitation, related to the enhanced PHA synthesis.

When comparing the different growth rates, approximately the same level of PHA synthesis was achieved for both conditions. Apart from a slightly increased ED pathway (2.4- and 1.3-fold under carbon and nitrogen-limitation, respectively) and TCA cycle (2.4- and 1.3-fold under carbon and nitrogen-limitation, respectively) at high dilution rate, no apparent differences were observed in carbon partitioning. The carbon flux through glyoxylate shunt and PHA synthesis remained almost unchanged.

### Potential for strain improvement of PHA-producing *P. putida* grown on glycerol

**Fig. 3:**
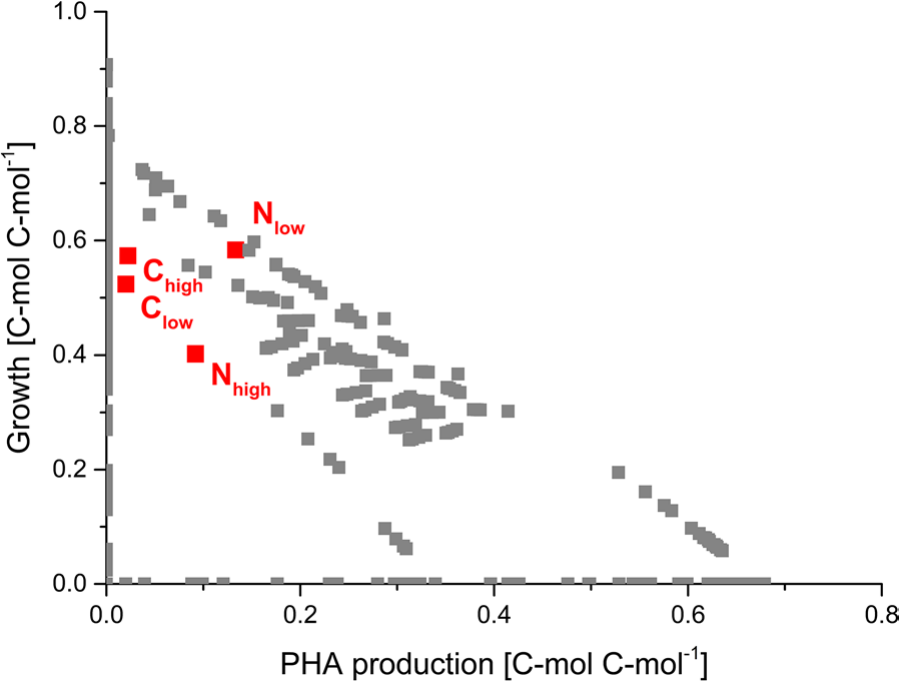
Solution space of the elementary flux mode analysis of glycerol-grown *P. putida*. Each *point* represents the biomass and the PHA yield of one unique non-decomposable pathway for *P. putida* grown on glycerol. All values are given in C-mol per C-mol. Additionally, the *red squares* display the values found in our chemostat experiments, where C and N are carbon and nitrogen-limited, respectively. The dilution rate is given as the index of C and N

To evolve into an economically viable candidate for industrial PHA production, *P. putida’s* productivity needs to be maximized. A first inspection is provided by integration of the achieved performance under the different conditions into the overall flux space (Fig. 3). Obviously, the glycerol-grown wild type accumulated significant amounts of the biopolymer. PHA synthesis was particularly efficient at low dilution rate under nitrogen limitation. Under these conditions, *P. putida* even approached the upper boundary of the feasible flux space. Ideally, PHA yield improvement would guide *P. putida* south eastbound, while staying close to the maximal efficiency border. However, even for this best scenario, *P. putida* spent most of the substrate for growth so that the obtained PHA yield is still far below the theoretical maximum, related to the best PHA-producing elementary flux mode (EFM). Although this extreme value at zero growth seems not reachable in practice and an achievable optimum will be realistically lower, the location of the experimental data in the flux space indicate a significant remaining optimization potential (Table 6).

**Table 6:** Maintenance coefficient of *P. putida* and other industrially relevant strains under aerobic conditions

Strain	Carbon source	Maintenance coefficient [mmol_substrate_·(gCDW·h)^−1^]	References
*P. putida KT2440*	*Glycerol*	*0.039*	This study
*A. aerogenes*	Glycerol	0.966	[46]
*K. aerogenes*	Glycerol	0.804	[46]
*E. coli* WT	Glycerol	0.627	[47]
*P. putida KT2440*	*Glucose*	*0.062*	[78]
*E. coli* MG1655	Glucose	0.370	[79]
*C. glutamicum*	Glucose	0.08	[52]
*B. subtilis*	Glucose	0.45	[80]
*B. subtilis*	Glucose	0.39	[51]
*K. aerogenes*	Glucose	0.350	[46]

**Fig. 4:**
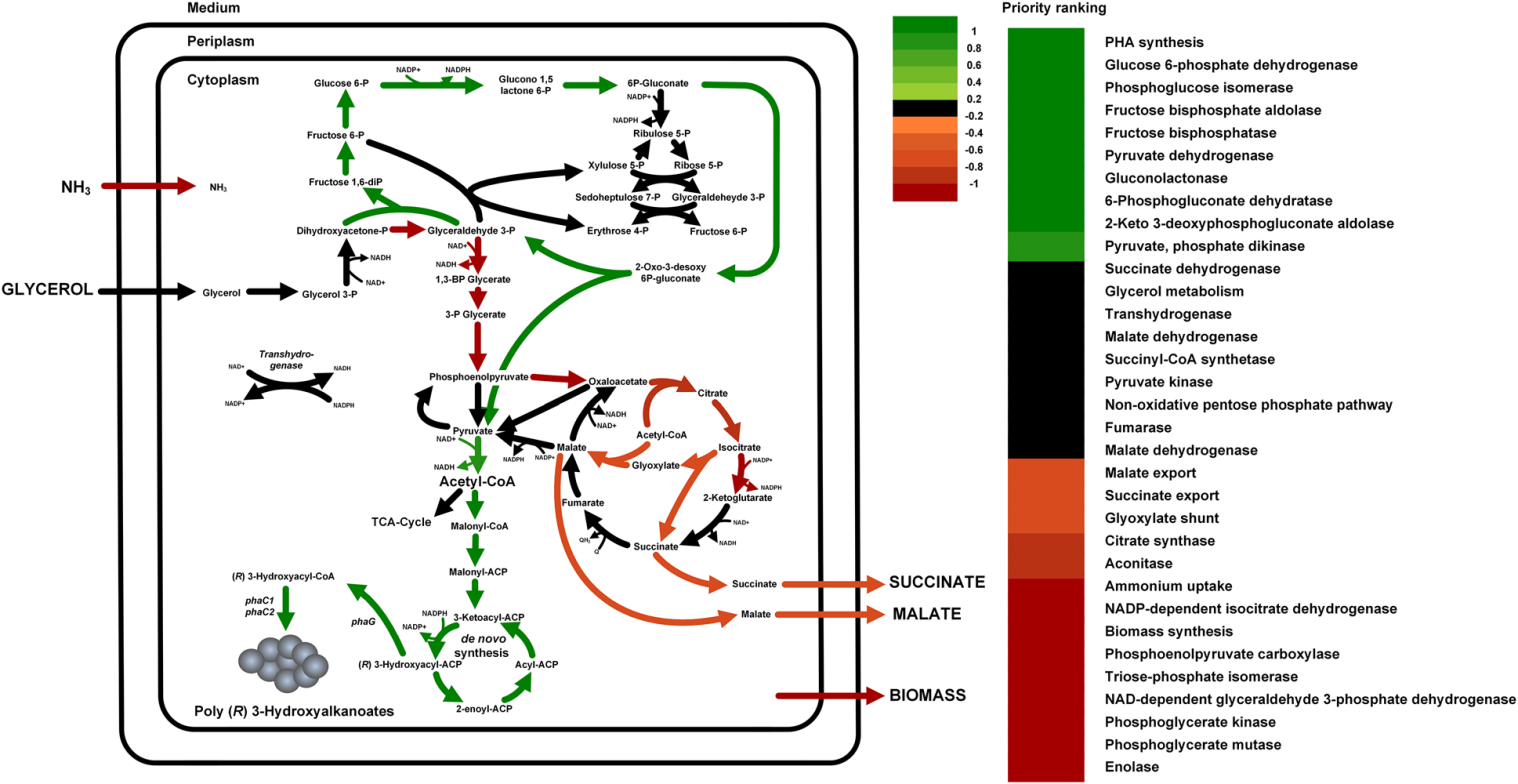
Genetic engineering targets towards improved PHA production in *P. putida* as predicted by an elementary flux mode-based correlation strategy (Flux Design). *Red arrows* represent reactions that are negatively correlated with PHA production and are therefore attenuation targets, whereas *green arrows* indicate positive correlation and thus overexpression targets. On the right, a priority ranking of all targets is given

Alongside improving culture conditions [16, 21], progress was recently achieved through rational strain engineering by flux design, an elementary flux mode-based correlation analysis for the prediction metabolic engineering targets, involving the full spectrum of amplification, attenuation, deletion and heterologous insertion [44, 45]. Applied here, calculation yielded 1533 unique EFM distributions that define the solution space for *P. putida* on glycerol (Fig. 3). Based on the cultivation data (Table 2), only the subset of modes that produced PHA, biomass, succinic acid and malic acid was selected for target prediction (Fig. 4). From these remaining modes, (i) a positive effect on PHA synthesis was identified for the ED pathway, (ii) the oxidative PP pathway and (iii) the first three reactions of the EMP pathway, as well as (iv) PHA production from pyruvate. Contrarily, a part of the TCA cycle, the glyoxylate shunt, the reactions of lower glycolysis between dihydroxyacetone phosphate and phosphoenolpyruvate and by-product synthesis had a negative influence on PHA synthesis, suggesting these reactions as targets for elimination and down-regulation, respectively.

## Discussion

### Low energy requirement of *P. putida* for maintenance enables versatile metabolic response

Impressively, the maintenance requirement of glycerol-grown *P. putida* KT440 is 16 times lower in comparison to *E. coli* and 20–25 times lower than that of other important strains grown on glycerol [46, 47] (Table 6). In addition, the maintenance coefficient was 1.5 times lower than that of *P. putida* growing on glucose and approximately ten times lower than that of several other glucose-grown industrial strains. As glycerol has a higher degree of reduction than glucose and produces twice as much reducing equivalents, when converted to phosphoenolpyruvate [48], it seems a straightforward substrate for the production of redox-demanding chemicals through fermentation processes. Moreover, glucose is not the preferred carbon source of strains belonging to the *Pseudomonas* genus [49, 50], making glycerol an attractive raw material for *P. putida* production processes. Several studies have attempted to reduce the carbon requirement for cell maintenance. Particularly, attention has been poured into industrial strains such as *Bacillus subtilis* [51], *Corynebacterium glutamicum* [52], and *Escherichia coli* [53]. Strains with a low maintenance coefficient can redirect more carbon towards a desired product, which is of utmost importance in white biotechnology as maintenance plays a key role under reduced growth rates [54]. Moreover, the maintenance requirement varies over time [54], thus having direct impact on the economics of the industrial process, particularly when operating the preferred fed-batch cultivation mode [55].

### The ED pathway is favored under both carbon and nitrogen limitation

As previously reported, nitrogen limitation promotes synthesis of PHA in *P. putida* [1]. A similar phenomenon has been observed for metabolically engineered *E. coli* strains able to accumulate PHB on glucose [56]. This modulation of the PHA flux has been related to several factors including (i) NADPH supply [57], (ii) activity of enzymes of the PHA pathway [56], and (iii) precursor availability [15, 58]. In this study, preferential use of the ED pathway and glyoxylate shunt was associated with increased PHA-production (Fig. 2). Particularly, recycling of resources through the upper EMP pathway, instead of channeling carbon directly through the lower EMP pathway, was associated with increased cofactor and PHA-precursor availability and thus a higher PHA biosynthetic flux. Conversely, this yielded less ATP, indicating that PHA production is constrained to a greater extent by cofactor and precursor availability than by ATP-deficiency. Additionally, the preferential use of the glyoxylate shunt, instead of the reductive TCA cycle between isocitrate and succinate, decreased the loss of carbon to CO_2_, however less reductive power could be generated. Therefore, it seems that PHA synthesis in *P. putida* under nitrogen-limitation is mainly constrained by precursor-availability. This also fits nicely with the predicted targets for improved PHA production (Fig. 4). The recycling of resources through the upper EMP pathway and ED pathway promotes PHA synthesis, whereas the reductive TCA-cycle is predicted to influence PHA synthesis adversely. In this regard, it is important to make a clear distinction among metabolic routes fueling PHA synthesis, as each of these pathways yields a specific amount of NADPH and PHA precursors. For instance, *C. necator* and metabolically engineered *E. coli* strains both possess a complete EMP and PP pathway. Additionally, the former has an active ED pathway, which works in conjunction with the EMP and PP pathway to yield pyruvate and acetyl-CoA. In this case, improved production of PHB has been achieved by re-directing the carbon flux through the PP pathway instead of the EMP pathway [59, 60]. As more NADPH is produced by the former strain, an increase in cofactor availability led to an improved synthesis of PHB. As *P. putida* does not have a complete EMP pathway [61], we have previously proven that overexpression of genes of the PP pathway does not improve PHA synthesis in *P. putida* KT2440, when grown on glucose [15]. On the contrary, our current findings point towards engineering of the ED pathway to improve PHA production capabilities in *P. putida*. This is also conform with the high natural flux through the ED pathway, when *P. putida* KT2440 is grown on glucose [62], and as shown here, also on glycerol (Fig. 2). Previous works have also pointed out the ED pathway as the possible main route when synthesizing mcl-PHAs on glycerol. A co-feeding strategy of glycerol and fatty acids was performed to evaluate its activity [24]. Here we fully confirm the use of the ED pathway by *P. putida* KT2440 under both carbon- and nitrogen-limiting conditions (Fig. 2) and propose its overexpression to improve PHA-productivity.

Evaluation of metabolic responses on different organizational levels is vital to understand an organisms’ survival and success in the environment [63]. Integration of the transcriptome and metabolic fluxes showed that, upon an increased dilution rate under carbon-limiting conditions, *P. putida* KT2440 exhibits a more active ED pathway and increased flux through the pyruvate node, associated with significant upregulation of pyruvate metabolism (PP0554, *acoA*) (Fig. 2a, b; Table 5). Also, catabolism and anabolism appear to be tightly coupled as no by-product (citrate, succinate, or malate) formation is found (Table 2). On the other hand, nitrogen-limiting growth drove a major flux of carbon via the hexose-phosphates to the ED pathway when the specific growth rate was increased (Fig. 2).

### Interconnection between transcripts and fluxes deciphers regulatory mechanisms of core carbon metabolism

#### Strong transcriptional regulation of glycerol metabolism

**Fig. 5:**
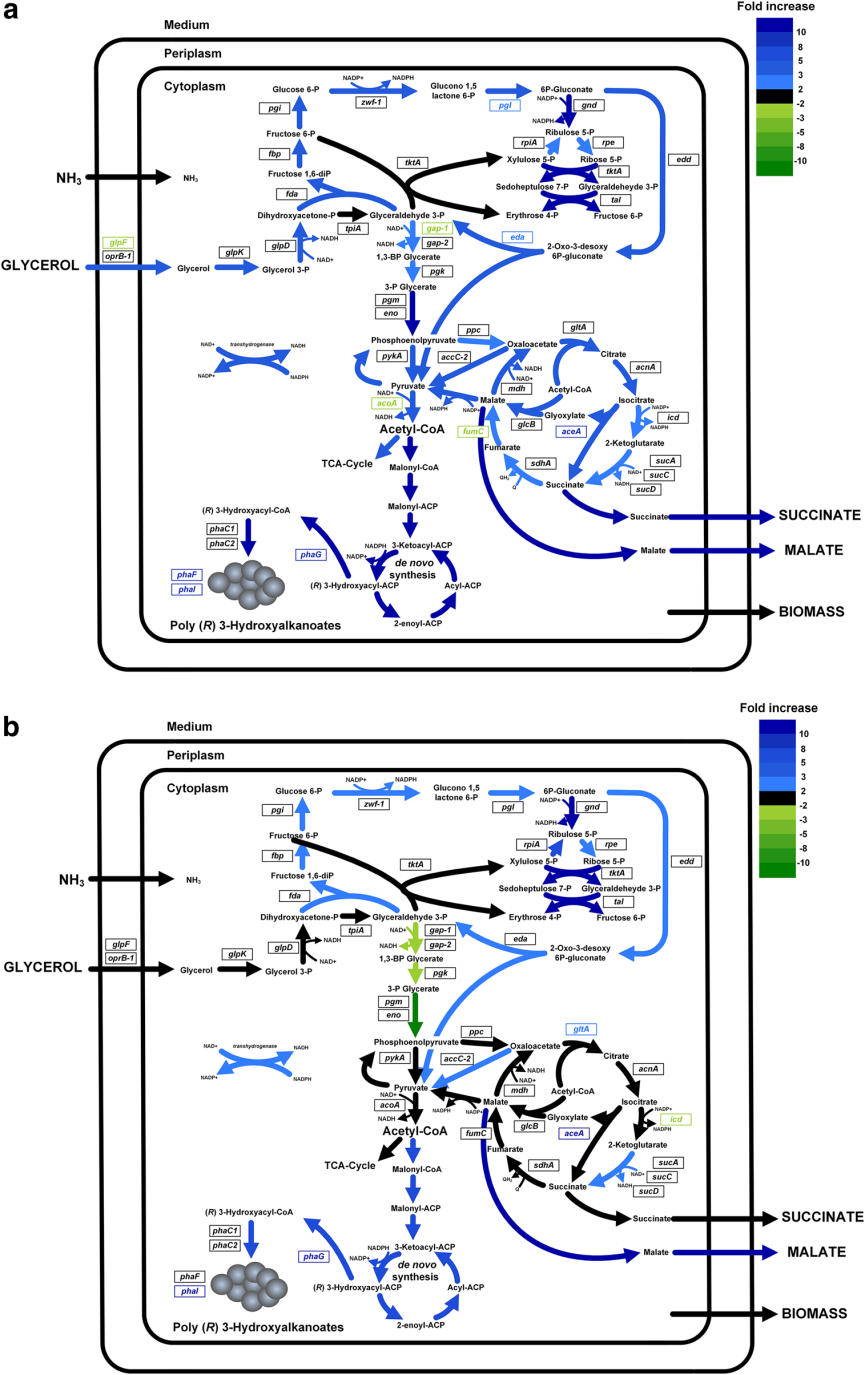
Comparison of fluxes and transcription levels between nitrogen and carbon-limitation under **a** low dilution rate and **b** high dilution rate. Significant flux differences are indicated by the *color of the arrow*, whereas differentially expressed genes are color-coded as gene names next to the respective *arrows*. *Green* represents transcription levels or fluxes that are significantly higher under carbon-limiting conditions. Contrastingly, *blue* indicated values that are increased under nitrogen-limiting conditions. Changes that exceeded a twofold increase or decrease were considered significant when the p value did not exceed 0.05

The shift from glycerol limitation to glycerol excess reveals unique flux and gene expression patterns in central carbon metabolism (Fig. 5). At low growth rate, the transcriptional regulator *glpR*, which represses genes, involved in the uptake and incorporation of glycerol in *P. putida* [24], did not show transcriptional changes, whereas the transporter *glpF* was transcriptionally attenuated (Table 5). It has previously been postulated that the presence of glycerol in the medium modulates the expression of *glpF* [24], which is consistent with our findings. Furthermore, at high growth rates the repression of *glpR*, mitigating the transcription of *glpF* and the regulator *araC* (PP1395) (Table 5), seems responsible for the decreased uptake rate of glycerol. Furthermore, at the transcript and flux level, a more active ED pathway, pyruvate node (anaplerotic reactions), and TCA cycle were found at a low dilution rate. In addition, pyruvate metabolism seems to be a key node, when glycerol is used as carbon source, as this pathway is transcriptionally modulated by the imposed nutrient limitation, a trait that has not been previously described for cells grown on glycerol under PHA-producing conditions (Table 5).

#### Complex regulation of isocitrate dehydrogenase mediated flux

Isocitrate dehydrogenase (*icd*, PP4012) showed no repression at low dilution rate, accompanied by a higher flux through its reaction (Fig. 5a), whereas at high dilution rate a significant decrease in transcription level was linked to a steady flux. Whilst previous studies have shown that at the mRNA level the gene *icd* is repressed under nitrogen-limiting conditions [9, 15], our findings indicate that regulation of the isocitrate dehydrogenase mediated flux might not be solely transcriptional. In fact, an increased enzymatic activity of Icd under nitrogen-limitation would explain the observed phenomenon.

#### Compelling correlation between PHA synthesis and expression of several genes from the PHA cluster

With regard to PHA synthesis, the shift from carbon- to nitrogen-limitation promoted accumulation of the biopolymer, whereby the *phaG* gene was most strongly up-regulated independently of the set dilution rate (Fig. 5). PhaG (transacylase) is the linking enzyme between de novo fatty acid synthesis and PHA biosynthesis in *Pseudomonas* strains [64, 65] and obviously supported enhanced PHA synthesis. In addition, PHA-granule forming enzymes, encoded by *phaI* and *phaF,* were up-regulated among the open reading frames of the PHA cluster, however, to a lesser extent (Table 5). They are known key elements of the PHA synthesis machinery, since they are involved in the segregation and distribution process of PHA [66–68]. Here, we discovered a direct correlation between strong synthesis of PHA and high expression of the *phaF* gene (Table 5). On the contrary, at a high specific growth rate, mRNA levels of *phaF* were unaffected, which could explain the observed low PHA production.

### PHA biosynthetic enzyme identified as potential bottleneck towards improved PHA production in *P. putida*

Transcriptome analysis revealed that pyruvate dehydrogenase (encoded by *acoA*) was overexpressed, when comparing cells growing at a high dilution rate against those at low dilution rate under nitrogen limitation (Table 5). This also correlates well with a high flux through this particular reaction (Fig. 2c, d). Nonetheless, when the carbon flux segregates from acetyl-CoA to various pathways (TCA cycle and PHA synthesis), the PHA flux was the same for both conditions (Fig. 2c, d). This leads to the hypothesis that PHA production in *P. putida* is restricted at the enzymatic level, probably at some point in the malonyl and/or the synthesis de novo fatty acid pathway. Additionally, the prediction of genetic targets for enhanced PHA synthesis by elementary mode correlation indicated that both the PHA biosynthetic pathways and the recycling of the ED pathway positively influence PHA productivity, as well as the elimination of by-product formation, being malate the target with the highest priority (Fig. 4).

## Conclusion

Overall, our results support the view that *P. putida* KT2440 has evolved to high metabolic versatility by a complex interplay of different molecular layers. This applies not only for the shift from carbon to nitrogen limitation, but also for a given specific growth rate, especially important, when cells are synthesizing PHAs. This study unravels that the Entner–Doudoroff and the glyoxylate pathways, and pyruvate metabolism play a key role when synthesizing mcl-PHA from glycerol as the only carbon and energy source. In addition, *P. putida* KT2440 modulates the expression of genes responsible for sensing its energetic state of the cell in order to satisfy the ATP requirement under PHA-producing conditions. Predictive metabolic modeling shows that there is still huge potential for improvement of mcl-PHA synthesis, where different metabolic engineering targets are proposed. In this way, genes belonging to the TCA cycle, ED pathway, and the synthesis of de novo fatty acids are identified as promising targets for genetic engineering towards improved PHA synthesis.

## Methods

### Strain

The wild-type *Pseudomonas putida* KT2440 (DSMZ, Braunschweig, Germany) was used in this study.

### Culture conditions

Cells were kept as frozen stock in 25 % (v/v) glycerol at −80 °C. To obtain single colonies, cells were plated onto Luria–Bertani agar plates. After 1 day incubation at 30 °C, liquid cultures were prepared by picking up a single colony from the plate and inoculating it into a 50 mL shake flask with 10 mL liquid M9 medium. M9 medium contained (per liter): 12.8 g Na_2_HPO_4_·7H_2_O, 3 g KH_2_PO4, 4.7 g (NH_4_)_2_SO_4_, 0.5 g NaCl, 0.12 g MgSO_4_·7H_2_O, 6.0 mg FeSO_4_·7H_2_O, 2.7 mg CaCO_3_, 2.0 mg ZnSO_4_·H2O, 1.16 mg MnSO_4_·H_2_O, 0.37 mg CoSO_4_·7H_2_O, 0.33 mg CuSO_4_·5H_2_O, 0.08 mg H_3_BO_3_, and 3 g glycerol as sole carbon source. Cells were grown under aerobic conditions at 30 °C in an orbital shaker (180 rpm, Innova, New Brunswik, NJ, USA). By taking a calculated volume of the overnight-grown cell suspension, cells were transferred into 500 mL baffled Erlenmeyer flasks with 100 mL M9 medium and cultivated as described above. This second pre-culture was used as an inoculum for the continuous process, operated in a lab scale bioreactor. The feed solution consisted of minimal medium (M9, described above) supplemented with 32.5 and 4.1 g L^−1^ of glycerol, for the nitrogen- and carbon-limited conditions, respectively.

### Continuous cultivation

Continuous cultivations were carried out under aerobic conditions with dilution rates ranging from 0.044 to 0.205 h^−1^ in a 2.0 L top-bench BIOSTAT B1 bioreactor (Sartorius B Systems GmbH, Melsungen, Germany) with a working volume of 1.0 L at 30 °C. The aeration rate was set to 0.4 L L^−1^ min^−1^ (PR4000, MKS instruments, Wilmington, MA, USA). The dissolved oxygen level was kept above 30 % air saturation by control of the agitation speed up to a maximum of 700 rpm. The pH was maintained at 7.0 ± 0.2 by automatic addition of 0.5 M H_2_SO_4_ and 1 M KOH, respectively. A gas analyzer, equipped with both a paramagnetic O_2_ and an infrared bench CO_2_ analyzer (Servomex Xentra 4100, Sugar Land, TX, USA), which recorded the concentration of carbon dioxide and oxygen, was coupled to the gas outlet of the bioreactor. The working volume of the fermenter was kept constant by removing the fermentation broth through a peristaltic pump and recording the weight with a balance, placed under the bioreactor.

### Analytics of substrates and products

Cell growth was recorded as optical density (OD) at 600_nm_ (Ultraspec 2000, Hitachi, Tokio, Japan). Cell dry weight was determined gravimetrically after collection of 10 mL culture broth (10 min, 4 °C, 8.000×*g*) in pre-weighed tubes, including a washing step with distilled water, and drying of the obtained pellet at 100 °C until constant weight. The ammonium concentration in cultivation supernatant was measured by a photometric test (LCK 303 kit, Hach Lange, Danaher, Washington, DC, USA). The concentration of glycerol and organic acids (succinate, formate, malate, citrate) in cultivation supernatant was analyzed by HPLC (Agilent 1260, Agilent, Krefeld, Germany), equipped with an 8 mm Rezex ROA-organic acid H column (Phenomenex, Torrance, CA, USA), operated with 0.013 N H_2_SO_4_ as mobile phase at 0.5 mL min^−1^ and 65 °C and detection using a refractive index detector (Agilent, Santa Clara, CA, USA).

### PHA characterization and quantification

Monomeric composition of PHA, as well as its cellular content, were determined by gas chromatography mass spectrometry (GC/MS) of the methanolyzed polyester. For this purpose, 10 mL culture broth was placed in a falcon tube and centrifuged (10 min, 4 °C, 9000×*g*), followed by a washing step with distilled water. The supernatant was discarded and the cell pellet was kept at −20 °C for further processing. Methanolysis was then carried out by re-suspending 5–10 mg of lyophilized aliquots in 2 mL chloroform and 2 mL methanol, containing 15 % (v/v) sulfuric acid and 0.5 mg mL^−1^ 3-methylbenzoic acid as internal standard, respectively, followed by incubation at 100 °C for 4 h. After cooling down to room temperature, 1 mL of demineralized water was added and the organic phase, containing the resulting methyl esters of the PHA monomers, was analyzed by GC–MS. Analysis was performed in a Varian 450GC/240MS ion trap mass spectrometer (Varian Inc., Agilent Technologies, Santa Clara, CA, USA) and operated by the software MS Workstation 6.9.3 (Varian Inc., Agilent Technologies). An aliquot (1 mL) of the organic phase was injected into the gas chromatograph at a split ratio of 1:10. Separation of compounds of interest, i.e. the methyl esters of 3-hydroxyexanoate, 3-hydroxyoctanoate, 3-hydroxydecanoate, 3-hydroxydodecanoate, 3-hydroxy-5-*cis*-dodecanoate, 3-hydroxytetradecanoate, was achieved by a FactorFour VF-5 ms capillary column (30 m × 0.25 mm i.d. × 0.25 mm film thickness, Varian Inc., Agilent Technologies). Helium was used as carrier gas at a flow rate of 0.9 mL min^−1^. Injector and transfer line temperature were 275 and 300 °C, respectively. The oven temperature program was: initial temperature 40 °C for 2 min, then from 40 °C up to 150 °C at a rate of 5 °C min^−1^ and finally up to 280 °C at a rate of 10 °C min^−1^. Positive ions were obtained using electron impact ionization at 70 eV and mass spectra were generated by scanning ions from *m/z* 50 to *m/z* 650. The PHA content (wt%) was defined as the percentage of the cell dry weight (CDW), represented by the polyhydroxyalkanoate.

### Transcriptome analysis

Aliquots of 10 mL culture broth were placed in RNAprotect buffer (Qiagen, Hilden, Germany) and centrifuged (1 min, 4 °C, 10,000×*g*). Cell pellets were frozen at −80 °C until further processing. Isolation of total RNA was performed using RNeasy kits (Qiagen, Venlo, The Netherlands), according to instructions by the manufacturer. Extracted RNA (2 μg) was labeled with either Cy3 or Cy5 using the ULS-system (Kreatech, Amsterdam, The Netherlands) according to the manufacturers manual. Equal amounts of Cy3 or Cy5-labelled RNA, one of them corresponding to the control and the other one to the condition to be analyzed, were mixed by pipetting. Labeled RNA (600 ng) was then fragmented and hybridized to the microarray. Agilent 8 × 15 K two-color microarrays (Agilent Technologies, Santa Clara, CA, USA), specifically designed for *P. putida* KT2440, was used for all transcriptional analyses. The microarrays were scanned using a GenePix Pro 4001 scanner and the GenePix 4.0 software (Axon Instruments, Foster City, CA, USA). Subsequent analysis of the microarrays was conducted with software packages (see below) from the Bioconductor suite. The image analysis results were read using the ‘limma’ packages [69]. The quality of the chips was analyzed with the ‘arrayQualityMetrics’ package [70]. Intensity values were background-corrected using the “‘normexp” method of the ‘limma’ package [71] and were normalized with the variance stabilization method [72]. Differentially expressed genes were identified by fitting the linear model (using the functions ‘lmFit’ and ‘eBayes’ from the ‘limma’ package [69]. Genes for which the adjusted p value (by Benjamini–Hochberg method) was lower than 0.05 and the fold change exceeded 2 in either direction were assumed to be differentially expressed.

### Metabolic network analysis

Flux balance analysis (FBA) was performed as described previously [73], using linear programming to maximize a chosen objective function, in our case growth. Shortly, FBA determines the optimal steady-state flux distribution in the metabolic network within a constraint space [74]. The constraints applied here, included experimentally determined uptake and production rates as well as PHA composition (Table 2).

Elementary flux modes (EFM’s) were calculated with efmtool, based on the null space approach and recursive enumeration with bit pattern trees [75]. The matrix, computed by the algorithm, comprises information on all thermodynamically and stoichiometrically possible pathways in the cell, which reduce metabolism into all feasible, unique, non-decomposable biochemical pathways [76]. Normalization of the EFM matrix and subsequent data interpretation was performed as described previously [44]. First, relative fluxes were normalized to the glycerol uptake flux. Subsequent flux correlation analysis investigated the target potential of individual metabolic reactions [44]. If the statistical significance of the correlation was met and the regression coefficient exceeded the cut-off of 0.7, the slope of the linear regression delivered the target potential coefficient to each individual reaction.

The metabolic network model, used for both analyses, was adapted from previous work [58]. Reactions for glycerol utilization, i.e. glycerol transport, glycerol dehydrogenase and dihydroxyacetone kinase, were implemented. Based on experimental observations, formation of gluconate was not considered. However, reactions for the secretion of succinate and malate were included (Table 2). PHA synthesis considered three different PHA types with different monomer composition. Stoichiometry of the reactions was based on the organism specific information provided in the Kyoto Encyclopedia of Genes and Genomes, i.e. the KEGG database [77] and is provided in the Appendix: Table 7. In total, the network consisted of 63 reactions, of which 18 described reversible conversions and 11 were transport reactions. Anabolic pathways for biomass synthesis were merged into a single equation. The precursor demand for growth was taken from previous work [58].

**Table 7:** Metabolic network model of *P. putida* KT2440

Pathway	Reactions
Transport reactions	‘→ GLY[e]’ ‘→ NH_3_[c]’ ‘→ SO_4_[c]’ ‘→ O_2_[c]’ ‘biomass[c] → ’ ‘FORMATE_ex[e] → ’ ‘MAL_ex[e] → ’ ‘SUCC_ex[e] → ’ ‘PHA[c] → ’ ‘ATPmaintenance[c] →’ ‘CO_2_[c] → ’
Glycerol uptake and conversion to glycerone-phosphate	‘GLY[e] → GLY[p]’ ‘GLY[p] → GLY[c]’ ‘GLY[c] + ATP[c → GLY-3P[c] + ADP[c]’ ‘GLY-3P[c] + NAD[c] ↔ DHAP[c] + NADH[c]’
Pentose phosphate pathway	‘G6P[c] + NADP[c] → 6-P-Gluconate[c] + NADPH[c]’ ‘6-P-Gluconate[c] + NADP[c] → RIB-5P[c] + CO_2_[c] + NADPH[c]’ ‘RIB-5P[c] ⟺ XYL-5P[c]’ ‘RIB-5P[c] ⟺ RIBO-5P[c]’ ‘S7P[c] + GAP[c] ⟺ RIBO-5P[c] + XYL-5P[c]’ ‘S7P[c] + GAP[c] ⟺ E4P[c] + F6P[c]’ ‘F6P[c] + GAP[c] ⟺ E4P[c] + XYL-5P[c]’
Entner-Doudoroff pathway	‘6-P-Gluconate[c] → KDPG[c]’ ‘KDPG[c] → GAP[c] + PYR[c]’
Embden-Meyerhof-Parnas pathway	‘G6P[c] ⟺ F6P[c]’ ‘FBP[c] → F6P[c]’ ‘FBP[c] ⟺ GAP[c] + DHAP[c]’ ‘DHAP[c] ⟺ GAP[c]’ ‘GAP[c] + NAD[c] ⟺ 13-PG[c] + NADH[c]’ ‘ADP[c] + 13-PG[c] ⟺ ATP[c] + 3-PG[c]’ ‘3-PG[c] ⟺ 2-PG[c]’ ‘2-PG[c] ⟺ PEP[c]’ ‘PEP[c] + ADP[c] → PYR[c] + ATP[c]’ ‘PYR[c] + NAD[c] → AcCoA[c] + NADH[c] + CO2[c]’ ‘PYR[c] + 2 ATP[c] → 2 ADP[c] + PEP[c]’
Citric acid cycle	‘AcCoA[c] + OAA[c] → CIT[c]’ ‘CIT[c] ↔ ICI[c]’ ‘ICI[c] + NADP[c] → AKG[c] + CO2[c] + NADPH[c]’ ‘AKG[c] + NAD[c] → SUCC-CoA[c] + NADH[c] + CO2[c]’ ‘SUCC-CoA[c] + ADP[c] ↔ SUCC[c] + ATP[c]’ ‘SUCC[c] + Q[c] ↔ FUM[c] + QH2[c]’ ‘FUM[c] ↔ MAL[c]’ ‘MAL[c] + NAD[c] ↔ OAA[c] + NADH[c]’
Organic acid production	‘MAL[c] → MAL_ex[e]’ ‘SUCC[c] → SUCC_ex[e]’ ‘FORMATE[c] → FORMATE_ex[e]’
Glyoxylate metabolism	‘ICI[c] → Glyoxy[c] + SUCC[c]’ ‘Glyoxy[c] + AcCoA[c] → MAL[c]’
Amphibolic metabolism	‘OAA[c] → PYR[c] + CO2[c]’ ‘PEP[c] + CO2[c] + ATP[c] → OAA[c] + ADP[c]’ ‘MAL[c] + NADP[c] → PYR[c] + NADPH[c] + CO2[c]’
PHA production	‘5 AcCoA[c] + 4 ATP[c] + 7 NADPH[c] → C10-PHA[c] + 4 ADP[c] + 7 NADP[c]’ ‘4 AcCoA[c] + 4 ATP[c] + 7 NADPH[c] → C8-PHA[c] + 4 ADP[c] + 7 NADP[c]’ ‘6 AcCoA[c] + 4 ATP[c] + 7 NADPH[c] → C12-PHA[c] + 4 ADP[c] + 7 NADP[c]’ ‘0.75 C10-PHA[c] + 0.17 C8-PHA[c] + 0.08 C12-PHA[c] → PHA[c]’
Energy metabolism	‘NADPH[c] + NAD[c] → NADP[c] + NADH[c]’ ‘(3) NADH[c] + (3) NADP[c] + ATP[c] → (3) NAD[c] + (3) NADPH[c] + ADP[c]’ ‘(0.5) O2[c] + NADH[c] + (1.33) ADP[c] → NAD[c] + (1.33) ATP[c]’ ‘(0.5) O2[c] + QH2[c] + (0.66) ADP[c] → Q[c] + (0.66) ATP[c]’ ‘ATP[c] → ADP[c] + ATPmaintenance[c]’ ‘SO4[c] + (3) NADPH[c] + (4) ATP[c] → H2S[c] + (3) NADP[c] + (4) ADP[c]’
Biomass production	‘(1.481) OAA[c] + (1.338) 3-PG[c] + (0.627) RIBO-5P[c] + (17.821) ATP[c] + (16.548) NADPH[c] + (6.965) NH3[c] + (3.548) NAD[c] + (2.930) AcCoA[c] + (2.861) PYR[c] + (1.078) AKG[c] + (0.361) E4P[c] + (0.72) PEP[c] + (0.233) H2S[c] + (0.072) F6P[c] + (0.206) G6P[c] + (0.129) GAP[c] → biomass[c] + (16.548) NADP[c] + (3.548) NADH[c] + (17.821) ADP[c] + (1.678) CO2[c]’

## Appendix

See Table 7.

### Deriving the ATP requirement for maintenance

Overall stoichiometry for synthesis of biomass constituents: (UK = unknown parameter)

$$UK_{1} \cdot CO_{2} + biomass - UK_{2} \cdot NADPH + UK_{3} \cdot NADH - Y_{xATP} \cdot ATP - UK_{4} \cdot CH_{{\frac{8}{3}}} O = 0$$

The required ATP and NADPH for biomass synthesis are supplied by the catabolic pathways:

1. Pentose phosphate pathway, where glycerol is completely oxidized to CO_2_
  $$CO_{2} + 2 \cdot NADPH + \frac{1}{3} \cdot NADH - \frac{1}{3} \cdot ATP - CH_{{\frac{8}{3}}} O = 0$$
2. ED pathway and TCA cycle, where glycerol is completely oxidized to CO_2_
  $$CO_{2} + \frac{1}{6} \cdot NADPH + \frac{13}{6} \cdot NADH + \frac{1}{3} \cdot ATP - CH_{{\frac{8}{3}}} O = 0$$
3. Oxidative phosphorylation
  $$P/O \cdot ATP - 0.5 O_{2} - NADH = 0$$

Consumption of ATP for maintenance

$$- ATP = 0$$

In matrix notation:

$$\left( {\begin{array}{*{20}c} { - UK_{4} } & 0 \\ { -1} & 0 \\ {-1} & 0 \\ 0 & {- 0.5} \\ 0 & 0 \\ \end{array} } \right) \left( {\begin{array}{*{20}c} {S_{Glycerol} } \\ {S_{{O_{2} }} } \\ \end{array} } \right) + \left( {\begin{array}{*{20}c} {UK_{1} } \\ 1 \\ {\begin{array}{*{20}c} 1 \\ 0 \\ 0 \\ \end{array} } \\ \end{array} } \right)\left( {P_{{CO_{2} }} } \right) + \left( {\begin{array}{*{20}c} 1 \\ {\begin{array}{*{20}c} 0 \\ 0 \\ {\begin{array}{*{20}c} 0 \\ 0 \\ \end{array} } \\ \end{array} } \\ \end{array} } \right)X + \left( {\begin{array}{*{20}c} {\begin{array}{*{20}c} { - Y_{xATP} } \\ { - \frac{1}{3}} \\ {\begin{array}{*{20}c} {\frac{1}{3}} \\ {P/O} \\ { - 1} \\ \end{array} } \\ \end{array} } & {\begin{array}{*{20}c} {\begin{array}{*{20}c} {UK_{3} } \\ {\frac{1}{3}} \\ {\begin{array}{*{20}c} {\frac{13}{6}} \\ { - 1} \\ 0 \\ \end{array} } \\ \end{array} } & {\begin{array}{*{20}c} { - UK_{2} } \\ {\frac{6}{3}} \\ {\begin{array}{*{20}c} {\frac{1}{6}} \\ 0 \\ 0 \\ \end{array} } \\ \end{array} } \\ \end{array} } \\ \end{array} } \right)\left( {\begin{array}{*{20}c} {X_{ATP} } \\ {\begin{array}{*{20}c} {X_{NADH} } \\ {X_{NADPH} } \\ \end{array} } \\ \end{array} } \right) = \left( {\begin{array}{*{20}c} 0 \\ {\begin{array}{*{20}c} 0 \\ 0 \\ {\begin{array}{*{20}c} 0 \\ 0 \\ \end{array} } \\ \end{array} } \\ \end{array} } \right)$$

With the rate vector of forward reaction rates:

$$v = \left( {\begin{array}{*{20}c} \mu \\ {\begin{array}{*{20}c} {v_{PP} } \\ {v_{ED} } \\ {\begin{array}{*{20}c} {v_{OP} } \\ {m_{ATP} } \\ \end{array} } \\ \end{array} } \\ \end{array} } \right)$$

The production and consumption of the three cofactors ATP, NADH and NADPH can now be balanced:

$$\left( {\begin{array}{*{20}c} 0 \\ {\begin{array}{*{20}c} 0 \\ 0 \\ {\begin{array}{*{20}c} 0 \\ 0 \\ \end{array} } \\ \end{array} } \\ \end{array} } \right) = \left( {\begin{array}{*{20}c} {\begin{array}{*{20}c} {\begin{array}{*{20}c} { - Y_{xATP} } & { - \frac{1}{3}} \\ {UK_{3} } & {\frac{1}{3}} \\ \end{array} } \\ {\begin{array}{*{20}c} { - UK_{2} } & {\frac{6}{3}} \\ \end{array} } \\ \end{array} } & {\begin{array}{*{20}c} {\begin{array}{*{20}c} {\frac{1}{3}} \\ {\frac{13}{6}} \\ {\frac{1}{6}} \\ \end{array} } & {\begin{array}{*{20}c} {P/O} \\ { - 1} \\ 0 \\ \end{array} } & {\begin{array}{*{20}c} { - 1} \\ 0 \\ 0 \\ \end{array} } \\ \end{array} } \\ \end{array} } \right)\left( {\begin{array}{*{20}c} \mu \\ {\begin{array}{*{20}c} {v_{PP} } \\ {v_{ED} } \\ {\begin{array}{*{20}c} {v_{OP} } \\ {m_{ATP} } \\ \end{array} } \\ \end{array} } \\ \end{array} } \right)$$

In addition to the three steady state balances above, we can also find equations for the specific glycerol and oxygen uptake and the CO_2_ production.

$$\left( {\begin{array}{*{20}c} {r_{Glycerol} } \\ {r_{{O_{2} }} } \\ \end{array} } \right) = \left( {\begin{array}{*{20}c} {UK_{4} \mu + v_{PP} + v_{ED} } \\ {0.5 \cdot v_{OP} } \\ \end{array} } \right)$$

$$\left( {r_{{CO_{2} }} } \right) = \left( {UK_{1} \mu + v_{PP} + v_{ED} } \right)$$

By elimation of v_PP_, v_ED_ and v_OP_ we get:

$$r_{Glycerol} = UK_{4} \cdot \mu + ( - 11) \cdot \frac{{ - Y_{xATP} + 2 \cdot UK_{2} + P/O \cdot UK_{3} + 13 \cdot UK_{2} \cdot P/O}}{{\frac{13}{3} + \frac{77}{3}P/O}} \cdot \mu - ( - 11) \cdot \frac{3}{13 + 77P/O} \cdot m_{ATP} + 6 \cdot UK_{2} \cdot \mu$$

$$r_{Glycerol} = a \cdot \mu + b = Y_{XS}^{true} \cdot \mu + m_{s}$$

where

$$m_{s} = \frac{33 }{13 + 77P/O}m_{ATP}$$

or

$$m_{ATP} = \left( {\frac{13}{33} + \frac{77}{33}P/O} \right)m_{s} .$$

## Abbreviations

D: dilution rate (h^−1^)
µ: specific growth rate (h^−1^)
q_glycerol_: specific glycerol uptake rate [g glycerol (g_CDW_)^−1^ h^−1^]
Y_x|glycerol_^true^: true biomass yield coefficient on glycerol (–)
m_glycerol_: maintenance coefficient on glycerol [mmol glycerol (g_CDW_)^−1^ h^−1^]
m_ATP_: ATP requirement for maintenance [(mmol ATP) (g_CDW_)^−1^ h^−1^]
P/O: phosphate:oxygen ratio (–)
